# Airway Wall Area Derived from 3-Dimensional Computed Tomography Analysis Differs among Lung Lobes in Male Smokers

**DOI:** 10.1371/journal.pone.0098335

**Published:** 2014-05-27

**Authors:** Nguyen Van Tho, Le Thi Huyen Trang, Yoshitaka Murakami, Emiko Ogawa, Yasushi Ryujin, Rie Kanda, Hiroaki Nakagawa, Kenichi Goto, Kentaro Fukunaga, Yuichi Higami, Ruriko Seto, Taishi Nagao, Tetsuya Oguma, Masafumi Yamaguchi, Le Thi Tuyet Lan, Yasutaka Nakano

**Affiliations:** 1 Division of Respiratory Medicine, Department of Medicine, Shiga University of Medical Science, Shiga, Japan; 2 Department of Medical Statistics, Shiga University of Medical Science, Shiga, Japan; 3 Health Administration Center, Shiga University of Medical Science, Shiga, Japan; 4 Respiratory Care Center, University Medical Center, Ho Chi Minh City, Vietnam; James Cook University, Australia

## Abstract

**Background:**

It is time-consuming to obtain the square root of airway wall area of the hypothetical airway with an internal perimeter of 10 mm (√Aaw at Pi10), a comparable index of airway dimensions in chronic obstructive pulmonary disease (COPD), from all airways of the whole lungs using 3-dimensional computed tomography (CT) analysis. We hypothesized that √Aaw at Pi10 differs among the five lung lobes and √Aaw at Pi10 derived from one certain lung lobe has a high level of agreement with that derived from the whole lungs in smokers.

**Methods:**

Pulmonary function tests and chest volumetric CTs were performed in 157 male smokers (102 COPD, 55 non-COPD). All visible bronchial segments from the 3^rd^ to 5^th^ generations were segmented and measured using commercially available 3-dimensional CT analysis software. √Aaw at Pi10 of each lung lobe was estimated from all measurable bronchial segments of that lobe.

**Results:**

Using a mixed-effects model, √Aaw at Pi10 differed significantly among the five lung lobes (R^2^ = 0.78, P<0.0001). The Bland-Altman plots show that √Aaw at Pi10 derived from the right or left upper lobe had a high level of agreement with that derived from the whole lungs, while √Aaw at Pi10 derived from the right or left lower lobe did not.

**Conclusion:**

In male smokers, CT-derived airway wall area differs among the five lung lobes, and airway wall area derived from the right or left upper lobe is representative of the whole lungs.

## Introduction

Computed tomography (CT) has been used as a non-invasive tool for assessing morphological changes in chronic obstructive pulmonary disease (COPD). The extent of emphysema and airway dimensions assessed using CT are associated with clinical symptoms and pulmonary function tests in patients with COPD [1]–[4]. Airway wall area assessed using quantitative CT is one index of airway dimensions that reflects airway remodeling [2], [3], [5], [6]. The square root of airway wall area of the hypothetical airway with an internal perimeter of 10 mm (√Aaw at Pi10) has been used as a comparable index of airway dimensions in several cross-sectional and longitudinal studies thanks to its adjustment for airway size [1], [5], [7]–[10]. In these studies, √Aaw at Pi10 of each patient was derived from all measured bronchial segments of the whole lungs. With the combination of volumetric CT scans and 3-dimensional (3-D) CT analysis software, the number of measured bronchial segments for each patient has increased dramatically [2], [11]–[13]. However, it is time-consuming to obtain √Aaw at Pi10 from all measured bronchial segments of the whole lungs, especially for studies with a large sample size.

It is not clear whether √Aaw at Pi10 is the same among the five lung lobes. Furthermore, it is not known whether √Aaw at Pi10 derived from each individual lobe is representative of the whole lungs. In this study, we hypothesized that √Aaw at Pi10 differs among the five lung lobes, and √Aaw at Pi10 derived from one certain lung lobe has a high level of agreement with that derived from the whole lungs in smokers. Some results of the present study have been reported in the form of abstracts [14], [15].

## Materials and Methods

### Ethics Statement

This study was conducted at the Outpatient Respiratory Clinic of University Medical Center in Ho Chi Minh City, Vietnam. The protocol of this study was approved by the Biomedical Ethics Committee of University Medical Center in Ho Chi Minh City. Written informed consent was obtained from all subjects.

### Study subjects

Subjects with COPD or without COPD (non-COPD) were recruited if they met all of the following criteria: male, age between 40 and 85 years, and former or current cigarette smoker with more than 10 pack-years of smoking. COPD was diagnosed according to the Global Initiative for Chronic Obstructive Lung Disease guidelines (GOLD): post-bronchodilator FEV_1_/FVC [ratio of forced expiratory volume in one second (FEV1) to forced vital capacity(FVC)] less than 70% [16]. Subjects were excluded if they had at least one of the following criteria: a history of asthma, COPD exacerbations within 6 weeks, chronic respiratory failure, contraindications to either CT or pulmonary function tests, and abnormalities on plain chest X-rays other than emphysema and/or minor linear opacities. Each subject underwent a complete medical interview, physical examination, pulmonary function tests, and chest CT on the same day.

### Pulmonary function tests

Patients performed standardized spirometry using the KoKo spirometer (nSpire Health Inc., Longmont, CO, USA) before and after inhaling 400 µg salbutamol (Ventolin, GlaxoSmithKline, Middlesex, UK). All maneuvers met American Thoracic Society/European Respiratory Society standards [17]. Post-bronchodilator parameters except for FEV_1_/FVC were expressed as percentage of predicted values based on the reference equations of NHANES III [18] with a correction factor of 0.88 [19]. Diffusing capacity of the lung for carbon monoxide (D_LCO_) was measured by the helium dilution and single-breath method using EasyOne Pro (ndd Medizintechnik AG, Zurich, Switzerland). D_LCO_% predicted was corrected for blood hemoglobin concentration.

### CT scanning protocol and 3-D CT analysis

All subjects were scanned using the same 64-slice CT scanner, Toshiba Aquilion 64 (Toshiba Corp., Tokyo, Japan), which was calibrated every day following the manufacturer's recommendations. Subjects were scanned from the apex to the bottom of the lungs with the CT scanning protocol of non-contrast, spiral mode, pitch 0.828, 120 kVp, 160 mA, rotation time 0.5 seconds, collimation 0.5 mm, and at suspended full inspiration. Before each CT scanning, the patients were coached how to deeply breathe in and hold their breath. CT images were reconstructed with 1-mm slice thickness, 0.5-mm interval, 320-mm field of view, 512×512 matrix, and standard reconstruction kernel (FC03 kernel). CT data were saved in the form of DICOM (Digital Imaging and Communications in Medicine) files and transferred to Shiga University of Medical Science for analysis. CT images were analyzed using Pulmonary Workstation 2 software (VIDA diagnostics, Coralville, IA, USA) (http://www.vidadiagnostics.com).

#### Emphysema analysis

From the original CT scan (Figure S1, Panel A), the right and left lungs and five lung lobes were automatically segmented (Figure S1, Panel B) [20], [21]. The observers edited the lung lobes when necessary. Emphysematous lesions were defined as voxels with CT attenuation less than −950 Hounsfield units (Figure S1, Panel D), so-called low attenuation volume (LAV), and expressed as the ratio of LAV to the corresponding lung volume (LAV%) [22]. The software automatically generated LAV% for the whole lungs and for each individual lobe.

#### Airway analysis

Segmental, sub-segmental, and sub-subsegmental bronchi were defined as the 3^rd^, 4^th^, and 5^th^ generations of bronchi, respectively. A bronchial segment was defined as a bronchial generation–from a parent branching point to the next child branching point. All visible bronchial segments from the 3^rd^ to 5^th^ generations were segmented, labeled, and measured using the software (Figures S2, Panels A and B) [13], [23]. For each bronchial segment, the software automatically measured the airway dimensions at every centerline voxel position along the middle third of that bronchial segment; the average of these measurements was the value of that bronchial segment. Because the software has been validated on physical phantoms with internal perimeter more than 6 mm [13] and majority of bronchial segments with the 3^rd^ generation have internal perimeter less than 20 mm, only bronchial segments with internal perimeters ranging from 6 mm to 20 mm (hereafter referred to as measurable bronchial segments) were selected for estimating √Aaw at Pi10. √Awa at Pi10 was calculated from the linear regression in which the square root of airway wall area of the measurable bronchial segment was plotted against its internal perimeter [5], [9]. √Aaw at Pi10 of the whole lungs was derived from all measurable bronchial segments of the whole lungs (Figure S2, Panel C), while √Aaw at Pi10 of each individual lobe was derived from all measurable bronchial segments of that lobe (Figure S2, Panel D).

Patients with noisy CT images, incomplete lobar fissures, or lung abnormalities other than emphysema were excluded from the statistical analysis. Further details about the 3-D CT analysis are provided in Supporting Information (Text S1).

### Statistical analysis

The differences in √Aaw at Pi10 or the number of measurable bronchial segments among the five lung lobes were examined using a mixed-effects model, in which subject was considered as a random effect and lung lobe as a fixed effect. The comparisons of √Aaw at Pi10 or the number of measurable bronchial segments between each pair of lobes were adjusted using the Tukey HSD method. The levels of agreement of √Aaw at Pi10 derived from each individual lobe with that derived from the whole lungs were evaluated using Bland-Altman plots, error range (measurement error x 1.96), and intra-class correlation coefficients (ICC) [24], [25]. The correlations between √Aaw at Pi10 or LAV% of each individual lobe and pulmonary function tests were evaluated using Spearman's correlation coefficients. A P-value < 0.05 was considered statistically significant. Statistical analysis was done using JMP 9.0.2 (SAS Institute Inc., Cary, NC) and IBM SPSS Statistics 20 (IBM Corp., Armonk, NY).

## Results

### Study population

Of 130 male smokers with COPD recruited, 28 were excluded from the statistical analysis due to noisy CT images (16 patients) and lung abnormalities (12 patients). Of 58 male smokers without COPD recruited, 3 were excluded due to noisy CT images (1 subject) and lung abnormalities (2 subjects). Therefore, 157 subjects with a total of 10,288 measurable bronchial segments were eligible for statistical analysis. Of 102 patients with COPD, 7 (6.9%) were in stage 1, 43 (42.2%) in stage 2, 43 (42.2%) in stage 3, and 9 (8.9%) in stage 4 according to the GOLD classification. Other clinical and pulmonary function characteristics of the 157 subjects are presented in Table 1.

**Table 1:** Clinical and pulmonary function characteristics of 157 male smokers.

Characteristics	Non-COPD (n = 55)	COPD (n = 102)	All subjects (n = 157)
Age (years)	53.9±7.1	65.6±10.0	61.5±10.7
Smoking history (pack-years)	29.7±11.3	39.2±14.5	35.9±14.2
Current smokers	50 (90.9%)	34 (33.3%)	84 (53.5%)
Former smokers	5 (9.1%)	68 (66.7%)	73 (46.5%)
Body mass index (kg/m^2^)	22.1±2.9	21.2±3.6	21.5±3.4
FVC (L)	3.52±0.48	2.69±0.80	2.98±0.81
FVC (% predicted)	97.1±13.3	78.4±17.6	85.0±18.5
FEV_1_/FVC (%)	81.5±6.0	45.7±10.7	58.2±19.5
FEV_1_ (L)	2.86±0.38	1.24±0.55	1.81±0.92
FEV_1_ (% predicted)	102.3±13.7	50.4±18.1	68.6±29.9
FEF_25–75%_ (L/s)	2.99±0.87	0.50±0.30	1.38±1.32
FEF_25–75%_ (% predicted)	105.2±29.0	21.1±11.3	50.6±44.7
D_LCO_ (mL/min/mmHg)*	21.15±4.05	13.18±5.73	17.86±6.20
D_LCO_ (% predicted)	81.7±14.4	56.7±20.2	71.4±20.9
D_LCO_/VA (mL/min/mmHg/L)	4.4±0.9	3.0±1.1	3.8±1.2
D_LCO_/VA (% predicted)	81.5±15.7	55.9±19.4	70.9±21.4

Data are presented as means ± SD or No. (%).

COPD, chronic obstructive pulmonary disease; COPD defined as post-bronchodilator FEV_1_/FVC < 70%; Non-COPD, subjects without COPD; D_LCO_, diffusing capacity of the lung for carbon monoxide; FEF_25–75%_, mean forced expiratory flow between 25% and 75% of forced vital capacity; FEV_1_, forced expiratory volume in one second; FVC, forced vital capacity; VA, alveolar volume.

*data from 54 non-COPD and 38 COPD subjects.

For each subject, the median number of measurable bronchial segments of the whole lungs was 64 (25^th^, 75^th^ percentiles 45.5, 82.5); the median √Aaw at Pi10 derived from the whole lungs was 3.79 mm (25^th^, 75^th^ percentiles 3.67, 3.93).

### √Aaw at Pi10 differs among the five lung lobes

**Figure 1:**
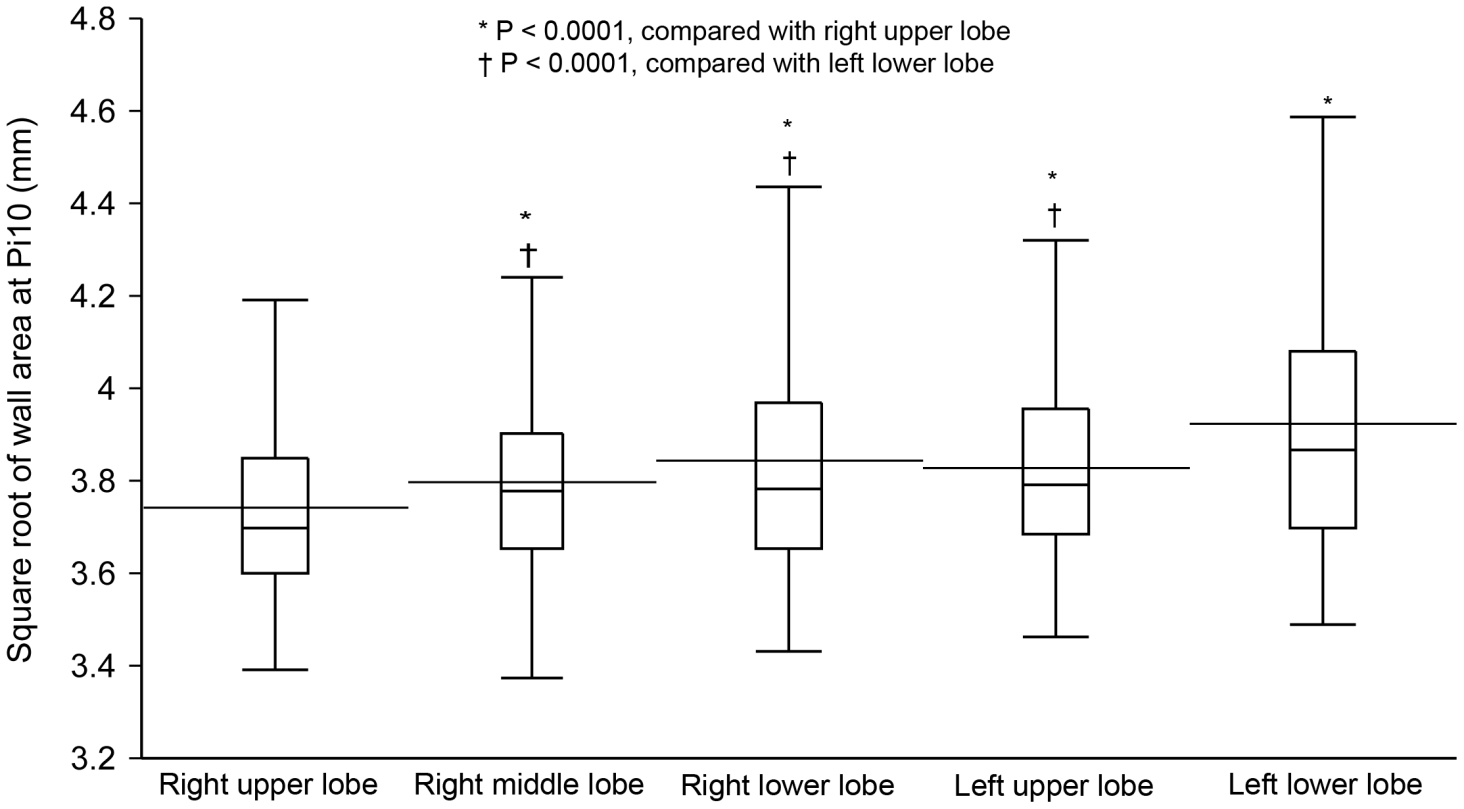
Comparison of √Aaw at Pi10 among the five lung lobes in 157 male smokers. √Aaw at Pi10, square root of airway wall area of the hypothetical airway with an internal perimeter of 10 mm; horizontal line inside the box, median; horizontal line crossing the box, mean; lower and upper margins of the box, 25^th^ and 75^th^ percentiles; whiskers, lines extend from the margins of the box to the lower and upper data point values. √Aaw at Pi10 differs significantly among the five lung lobes (R^2^ = 0.78, P<0.0001 by a mixed-effects model). **√**Aaw at Pi10 of the right upper lobe is significantly thinner than that of the four other lobes; **√**Aaw at Pi10 of the left lower lobe is significantly thicker than that of the other lobes; there are no significant differences in **√**Aaw at Pi10 between each pair of lobes among the right middle, right lower, and left upper lobes.

√Aaw at Pi10 differed significantly among the five lung lobes (R^2^ = 0.78, P<0.0001 by a mixed-effects model; Table 2). Among these lobes, √Aaw at Pi10 of the right upper lobe (RUL) was significantly thinner than that of the four other lobes; √Aaw at Pi10 of the left lower lobe (LLL) was significantly thicker than that of the other lobes (Figure 1). There were no differences in √Aaw at Pi10 between pairs of lobes among the right middle lobe (RML), right lower lobe (RLL), and left upper lobe (LUL). The magnitude of the difference in √Aaw at Pi10 between each pair of lobes was greater in COPD than in non-COPD subjects (Table 3).

**Table 2:** Comparisons of computed tomography characteristics among the five lung lobes in 157 male smokers.

Lung lobe	√Aaw at Pi10 (mm)†	Number of measurable bronchial segments‡	LAV (%)
Right upper	3.70 (3.60, 3.85)	13 (10, 16)	0.4 (0.1, 4.1)
Right middle*	3.78 (3.65, 3.90)	7 (5, 9)	0.2 (0.1, 1.3)
Right lower	3.78 (3.65, 3.97)	18 (12, 25)	0.2 (0.1, 1.8)
Left upper	3.79 (3.69, 3.95)	14 (10, 18)	0.5 (0.1, 4.0)
Left lower	3.87 (3.70, 4.08)	12 (9, 18)	0.2 (0.1, 2.4)

Data are presented as medians (25^th^, 75^th^ percentiles). LAV, low attenuation volume at the threshold level of −950 Hounsfield units; √Aaw at Pi10, square root of airway wall area of the hypothetical airway with an internal perimeter of 10 mm.

*data from 146 subjects.

† P<0.0001 for comparison among the five lung lobes by a mixed-effects model (R^2^ = 0.78).

‡ P<0.0001 for comparison among the five lung lobes by a mixed-effects model (R^2^ = 0.75).

**Table 3:** Magnitude of the difference in √Aaw at Pi10 between pair of lobes in non-COPD and COPD subjects.

Difference between pair of lobes	Non-COPD (n = 55)	COPD (n = 102)
LLL – RUL (mm)	0.10 (0.06, 0.15)	0.22 (0.17, 0.27)
LLL – RML (mm)	0.05 (0.00, 0.09)	0.14 (0.08, 0.19)
LLL – RLL (mm)	0.05 (0.00, 0.10)	0.09 (0.04, 0.14)
LLL – LUL (mm)	0.02 (−0.03, 0.07)	0.14 (0.09, 0.19)
RML – RUL (mm)	0.06 (0.01, 0.11)	0.08 (0.03, 0.14)
RLL – RUL (mm)	0.05 (0.00, 0.10)	0.13 (0.08, 0.18)
LUL − RUL (mm)	0.08 (0.04, 0.13)	0.09 (0.04, 0.14)

Data are presented as mean differences (95% CI).

√Aaw at Pi10, square root of airway wall area of the hypothetical airway with an internal perimeter of 10 mm; RUL, right upper lobe; RML, right middle lobe; RLL, right lower lobe; LUL, left upper lobe; LLL, left lower lobe.

The difference in √Aaw at Pi10 between each pair of lobes was examined using a mixed-effects model and adjusted by the Tukey HSD method. The magnitude of the difference in √Aaw at Pi10 between each pair of lobes is greater in COPD than in non-COPD subjects.

The number of measurable bronchial segments was greatest in RLL (P<0.0001 for all comparisons between RLL with each of the remaining lobes) and fewest in RML (P<0.0001 for all comparisons between RML with each of the remaining lobes) (Table 2). However, the number of measurable bronchial segments was not different between RUL and LUL (P = 0.0898), between RUL and LLL (P = 0.9068), or between LUL and LLL (P = 0.4745).

### √Aaw at Pi10 derived from the upper lobes is representative of the whole lungs

**Figure 2:**
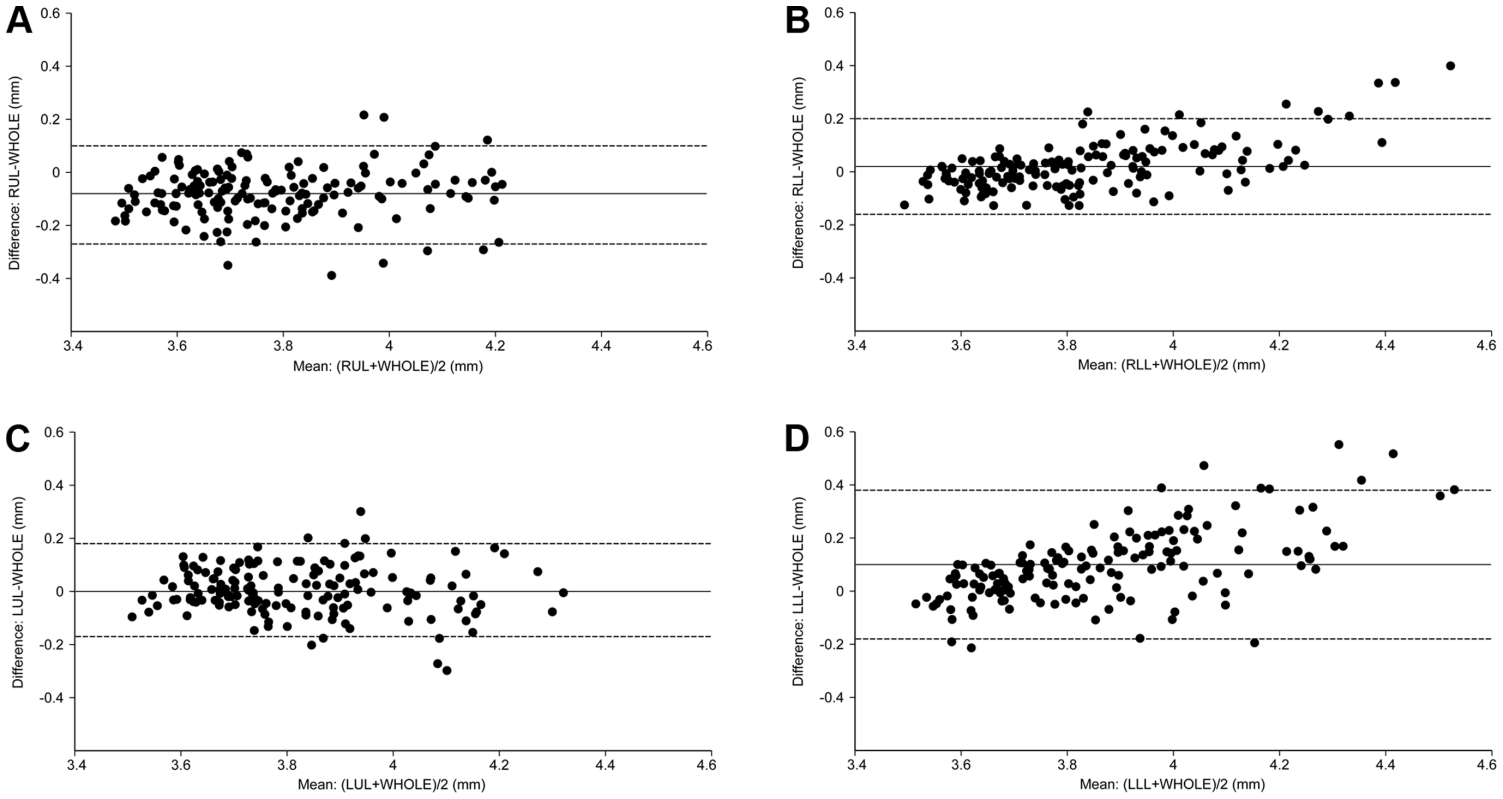
Levels of agreement of √Aaw at Pi10 between each individual lobe and the whole lungs. √Aaw at Pi10, square root of airway wall area of the hypothetical airway with an internal perimeter of 10 mm; the solid line is the mean difference; the dashed lines are the upper and lower limits of agreement. Comparisons of **√**Aaw at Pi10 derived from the whole lungs (WHOLE) with that derived from the right upper lobe (RUL) (Panel A), right lower lobe (RLL) (Panel B), left upper lobe (LUL) (Panel C), or left lower lobe (LLL) (Panel D). Measurement error scatters evenly around the line of mean difference in RUL and LUL and unevenly in RLL and LLL (the difference increases in proportion to the levels of the means).

The Bland-Altman plots show that the measurement error was scattered evenly around the line of mean difference in RUL and LUL, but unevenly in RLL and LLL (Figure 2). The magnitude of the difference increased when the mean of √Aaw at Pi10 increased in RLL and LLL (Kendall's correlation coefficient between the differences and the means, τ = 0.37, P<0.0001 and τ = 0.44, P<0.0001, respectively), which means that √Aaw at Pi10 derived from RLL or LLL had a systematic bias compared with that derived from the whole lungs. The error range was wider and the ICC was lower in RML than in RUL or LUL (Table 4).

**Table 4:** Levels of agreement of √Aaw at Pi 10 between each individual lobe and the whole lungs in 157 male smokers.

Lung lobe	Mean of the difference (mm)	Limits of agreement (mm)	Error range (mm)	ICC
Right upper	−0.08	−0.27, 0.10	0.13	0.937
Right middle*	−0.01	−0.27, 0.25	0.18	0.865
Right lower	0.02	−0.16, 0.20	0.13	0.955
Left upper	0.00	−0.17, 0.18	0.13	0.936
Left lower	0.10	−0.18, 0.38	0.20	0.899

√Aaw at Pi10, square root of airway wall area of the hypothetical airway with an internal perimeter of 10 mm; ICC, intra-class correlation coefficient.

^*^data from 146 subjects.

√Aaw at Pi10 derived from the right upper lobe, right lower lobe, or left upper lobe has a narrower error range and a higher ICC than that derived from the right middle lobe or left lower lobe.

From Figure 2 and Table 4, √Aaw at Pi10 derived from RUL or LUL should be selected as a surrogate for that of the whole lungs. One can estimate √Aaw at Pi10 of the whole lungs from that of RUL (by adding 0.08 mm) or LUL (without adjustment). If one estimates √Aaw at Pi10 of the whole lungs from that of RUL, 93.7% of the variation in √Aaw at Pi10 results from the true variation between subjects and 6.3% is attributed to the measurement error associated with RUL; 95% of the estimate values lie within the range of 0.13 mm above and 0.13 mm below the actual √Aaw at Pi10. If one estimates √Aaw at Pi10 of the whole lungs from that of LUL, 93.6% of the variation in √Aaw at Pi10 results from the true variation between subjects and 6.4% is attributed to the measurement error associated with LUL; 95% of the estimate values lie within the range of 0.13 mm above and 0.13 mm below the actual √Aaw at Pi10.

### CT measures from each lobe correlate with pulmonary function tests

√Aaw at Pi10 derived from each individual lobe or the whole lungs was negatively associated with FEV_1_/FVC, FEV_1_% predicted, and FEF_25–75%_% predicted (mean forced expiratory flow between 25% and 75% of forced vital capacity) (Table 5). However, the correlation coefficients were lower in RUL than in RLL, LUL, or LLL. The scatter plots of the relationship between √Aaw at Pi10 derived from RUL or LUL and FEV_1_/FVC or FEV_1_% predicted are presented in Figure S3 (Supporting Information). LAV% derived from each individual lobe or the whole lungs was also negatively associated with FEV_1_/FVC, FEV_1_% predicted, and D_LCO_% predicted (Table 6).

**Table 5:** Correlations between √Aaw at Pi10 and pulmonary function tests in 157 male smokers.*

√Aaw at Pi10 (mm)	FEV_1_/FVC (%)	FEV_1_ (% predicted)	FEF_25–75%_ (% predicted)
Right upper lobe	−0.55	−0.50	−0.57
Right middle lobe†	−0.52	−0.53	−0.53
Right lower lobe	−0.60	−0.58	−0.62
Left upper lobe	−0.62	−0.58	−0.62
Left lower lobe	−0.60	−0.57	−0.63
Whole lungs	−0.66	−0.63	−0.68

Data are presented as Spearman ρ correlation coefficients.

Abbreviations as in Tables 1 and 2.

*P<0.0001 for all correlation coefficients.

† data from 146 subjects.

**Table 6:** Correlations between LAV% and pulmonary function tests in 157 male smokers.*

LAV (%)	FEV_1_/FVC (%)	FEV_1_ (% predicted)	D_LCO_ (% predicted)†
Right upper lobe	−0.65	−0.58	−0.56
Right middle lobe‡	−0.59	−0.52	−0.46
Right lower lobe	−0.66	−0.65	−0.64
Left upper lobe	−0.72	−0.63	−0.60
Left lower lobe	−0.59	−0.60	−0.63
Whole lungs	−0.72	−0.67	−0.67

Data are presented as Spearman ρ correlation coefficients.

Abbreviations as in Tables 1 and 2.

*P<0.0001 for all correlation coefficients.

† data from 92 subjects.

‡ data from 146 subjects.

## Discussion

This study shows that √Aaw at Pi10 differed among the five lung lobes in male smokers: it was thinnest in RUL and thickest in LLL (Figure 1). This study also shows that √Aaw at Pi10 derived from RUL or LUL had a high level of agreement with that derived from the whole lungs, while √Aaw at Pi10 derived from RLL or LLL did not (Figure 2).

The difference in √Aaw at Pi10 among the five lung lobes may result from the effect of cardiac motion artifact, the heterogeneity of airway wall remodeling in smokers, or a combination of both factors. The cardiac motion artifact makes the airway wall blurrier or thicker than it is. Because it has the greatest effect on LLL, whose measurable bronchial segments are located closest to the left ventricle, √Aaw at Pi10 of LLL was thickest among the five lung lobes (Figure 1). The airway wall remodeling may be heterogeneous in smokers. This is supported by the finding that the magnitude of the difference in √Aaw at Pi10 between each pair of lobes was greater in COPD than in non-COPD subjects (Table 3). This finding also extends similar findings in asthma and healthy subjects. Aysola et al. (2008) found that wall area percent was significantly different among segmental bronchi in severe asthma, mild-to-moderate asthma, and healthy subjects. The magnitude of the difference was significantly greater in severe asthma than in the two remaining groups [26]. However, Ohara et al. (2006) found that wall area percent of the apical segmental bronchus of RUL was not significantly different from that of the basal segmental bronchus of RLL in 30 male patients with COPD [27]. The reasons for no difference in that study may include: a sample size not large enough to detect the difference; internal perimeter of the measured bronchi not controlled; and only two segmental bronchi measured and compared.

The position of the lung lobes may determine the levels of agreement of √Aaw at Pi10 between each individual lobe and the whole lungs. √Aaw at Pi10 derived from the upper lobes had a high level of agreement with that derived from the whole lungs because of a small and consistent bias (Figure 2, Panels A and C). All (for RUL) or most (for LUL) measurable bronchial segments from these two lobes are not affected by the cardiac motion artifact. The high level of agreement of √Aaw at Pi10 between RUL and the whole lungs in the present study justifies the assumption of a previous study [3] that airway wall percent of the RUL apical segmental bronchus could be representative of the whole lungs in male smokers. In contrast, √Aaw at Pi10 derived from the lower lobes did not have a high level of agreement with that derived from the whole lungs because of the systematic bias (Figure 2, Panels B and D). The measurable bronchial segments of RLL or LLL are not measured under the same condition due to the effect of cardiac motion artifact–the magnitude of the effect is greater for thicker airways. Therefore, subjects with thicker measurable bronchial segments in the lower lobes would have √Aaw at Pi10 derived from the lower lobes greater than that derived from the whole lungs and vice versa.

The different number of measurable bronchial segments among lung lobes may not result in the different levels of agreement of √Aaw at Pi10 between each individual lobe and the whole lungs. Both RUL and LUL had significantly fewer measurable bronchial segments than RLL (Table 2), but the level of agreement of both upper lobes was higher than that of RLL (Figure 2).

The finding that √Aaw at Pi10 or LAV% derived from each individual lobe correlated well with pulmonary function tests (Tables 5 and 6) again supports the use of CT measures of one lobe as surrogates for those of the whole lungs. Because we edited the lung lobes when necessary for each subject, the inherent error of LAV% calculated from each lobe is negligible. The correlation coefficients from RUL were lower than those from RLL, LUL, or LLL (Tables 5 and 6) because the lung volume is smaller in RUL than in each of the three other lobes; pulmonary function tests are contributed to by other factors, which are also different among lobes, besides airway wall area and emphysema [28]. The associations between √Aaw at Pi10 or LAV% of the whole lungs and pulmonary function tests (Tables 5 and 6) are in accordance with findings from the previous studies [3], [29].

To the best of our knowledge, this is the first study to compare √Aaw at Pi10 among the five lung lobes in male smokers using 3-D CT analysis software to measure all visible airways from the 3^rd^ to 5^th^ generations. The difference in airway wall area among the five lung lobes suggests that airway dimensions are heterogeneous in smokers, especially in those with COPD. This finding also suggests that lung lobes should be taken into account when estimating airway dimensions from CT images for each subject, or when comparing airway dimensions between or within subjects. Therefore, there are two options to estimate √Aaw at Pi10 for each subject: from all measurable bronchial segments of the whole lungs or from all measurable bronchial segments of the lobe that is representative of the whole lungs. The first option is usually very time-consuming and sometimes yields inconsistent estimates because of the variable effect of cardiac motion artifact on measurable bronchial segments of the lower lobes–the magnitude of the effect depends on the cardiac cycle when the lower lobes are being scanned. The second option may be the alternative to save time and to generate a consistent estimate of √Aaw at Pi10 in studies with a large sample size, especially in longitudinal studies. Based on the results of the present study, we recommend that √Aaw at Pi10 derived from RUL or LUL can be used as a surrogate for that derived from the whole lungs.

This study has some limitations. First, it was done at only one institution with one CT scanning protocol. Indices of the agreement test may change when the method of estimating √Aaw at Pi10 from each individual lobe is applied to another population with different inclusion criteria. However, the measurement error from the upper lobes is still consistent because the effect of cardiac motion artifact on the measurable bronchial segments of the upper lobes, especially RUL, is negligible. Second, since only male smokers were included in this study, one should be cautious to generalize the findings to female smokers. Third, √Aaw at Pi10 was not measured directly from the software, but calculated from the linear regression equation. Some argue that the regression equation does not explain most of the variation of √Aaw at Pi10 calculated from each individual lobe. However, the coefficient of determination of the regression analysis from each individual lobe was not lower than that from the whole lungs (Table S1). Finally, because airway dimensions were quantified using only one available method of airway analysis, we do not know whether the findings would be reproducible when a new method of airway analysis is applied [30].

In conclusion, airway wall area derived from 3-D CT analysis differs among the five lung lobes, and airway wall area derived from RUL or LUL is representative of the whole lungs in male smokers.

## Supporting Information

Figure S1 **Emphysema analysis using Pulmonary Workstation 2.** (A) An underlying coronal CT section of a patient with chronic obstructive pulmonary disease stage 3. (B) The CT section is masked with different colors after lobe segmentation: brown, right upper lobe; purple, right middle lobe; yellow, right lower lobe; green, left upper lobe; and blue, left lower lobe. (C) All pixels with CT attenuation less than −950 Hounsfield units are masked with different colors depending on lung lobes in the same CT section as in Panel A. (D) Emphysema extent quantified by the “density mask” method in a 3-D CT image: the size of the “balls” represents the size of emphysematous lesions that are connected voxels with CT attenuation less than −950 Hounsfield units. Emphysematous lesions are masked with different colors depending on the lung lobes.
(TIF)

Figure S2 **Airway analysis using Pulmonary Workstation 2.** (A) The 3-D bronchial tree is segmented, and one bronchial pathway is labeled until the 5^th^ generation (RMB, right main bronchus; BronInt, bronchus intermedius; RLL, right lower lobe basal bronchus; RB8, right basal anterior segmental bronchus–the 3^rd^ generation; RB8b, right basal anterior sub-segmental bronchus–the 4^th^ generation; RB8bi, right basal anterior sub-subsegmental bronchus–the 5^th^ generation). (B) One bronchial pathway is reconstructed as a straightened airway image. The yellow dash line indicates where a 2-D slice, which is perpendicular to the centerline, is resampled for measuring airway dimensions at a centerline voxel position. (C) The square root of airway wall area of the hypothetical airway with an internal perimeter of 10 mm (√Aaw at Pi10) is derived from all measurable bronchial segments of the whole lungs of a representative COPD patient. (D) √Aaw at Pi10 is derived from all measurable bronchial segments of the right upper lobe of the same patient as in Panel C.
(TIF)

Figure S3 **Correlations between √Aaw at Pi10 derived from the upper lobes and FEV_1_/FVC or FEV_1_% predicted.** √Aaw at Pi10, square root of airway wall area of the hypothetical airway with an internal perimeter of 10 mm; FVC, forced vital capacity; FEV_1_, forced expiratory volume in one second. √Aaw at Pi10 derived from the right upper lobe is negatively associated with FEV_1_/FVC (Panel A) and FEV_1_% predicted (Panel B). Similarly, √Aaw at Pi10 derived from the left upper lobe is negatively associated with FEV_1_/FVC (Panel C) and FEV_1_% predicted (Panel D).
(TIF)

Table S1 **Coefficients of determination (R^2^) of the linear regression analyses for √Aaw at Pi10.**
(DOC)

Text S1 **Details about the 3-D CT analysis using Pulmonary Workstation 2 software.**
(DOC)
