# Supplementary figures and images for: Airway Wall Area Derived from 3-Dimensional Computed Tomography Analysis Differs among Lung Lobes in Male Smokers
Source: PLoS One. 2014 May 27;9(5):e98335. doi: 10.1371/journal.pone.0098335 (PMC4035347; doi:10.1371/journal.pone.0098335)

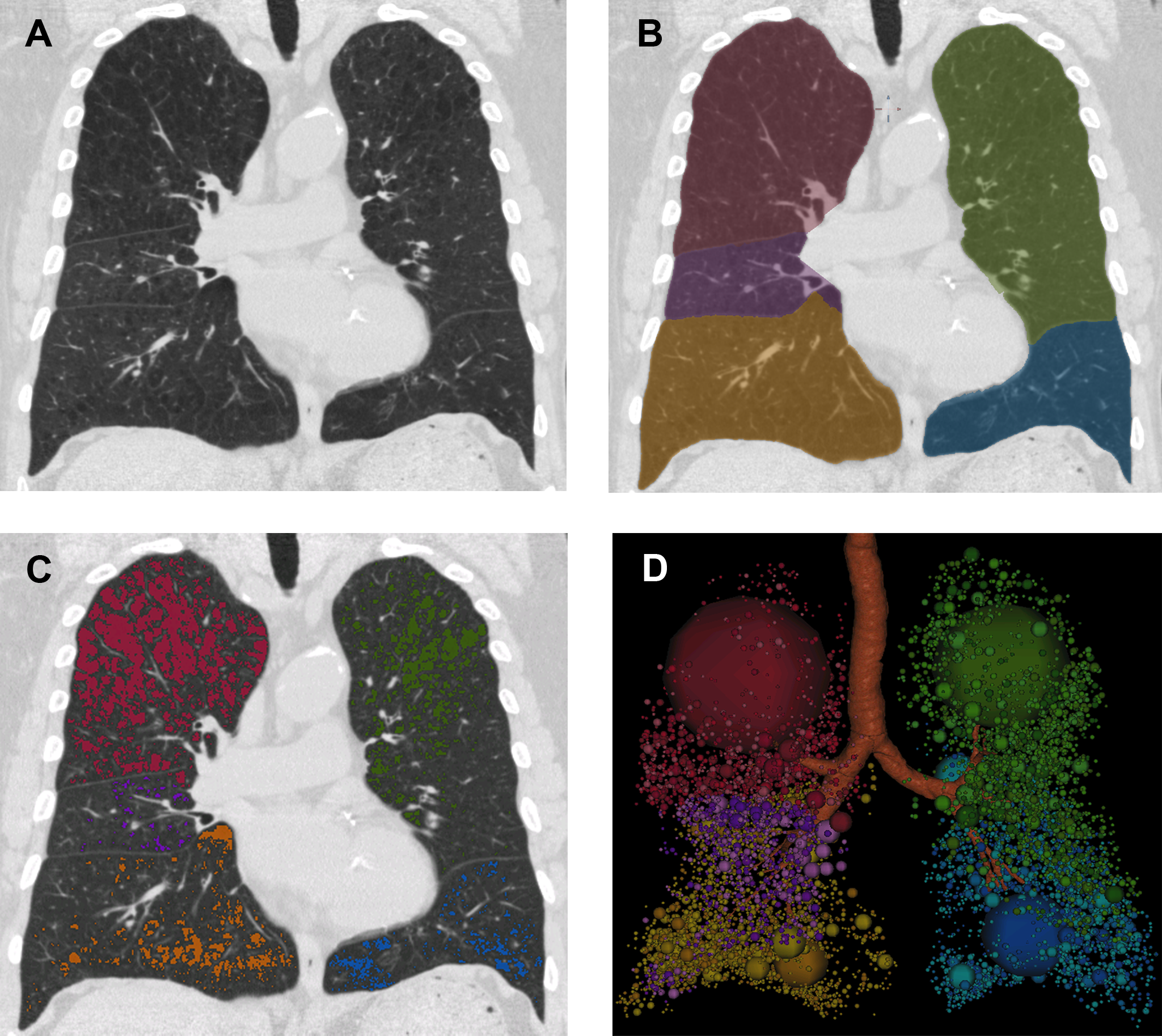

Supplement: Figure S1 — Emphysema analysis using Pulmonary Workstation 2. (A) An underlying coronal CT section of a patient with chronic obstructive pulmonary disease stage 3. (B) The CT section is masked with different colors after lobe segmentation: brown, right upper lobe; purple, right middle lobe; yellow, right lower lobe; green, left upper lobe; and blue, left lower lobe. (C) All pixels with CT attenuation less than −950 Hounsfield units are masked with different colors depending on lung lobes in the same CT section as in Panel A. (D) Emphysema extent quantified by the “density mask” method in a 3-D CT image: the size of the “balls” represents the size of emphysematous lesions that are connected voxels with CT attenuation less than −950 Hounsfield units. Emphysematous lesions are masked with different colors depending on the lung lobes. (TIF) [file pone.0098335.s001.tif]

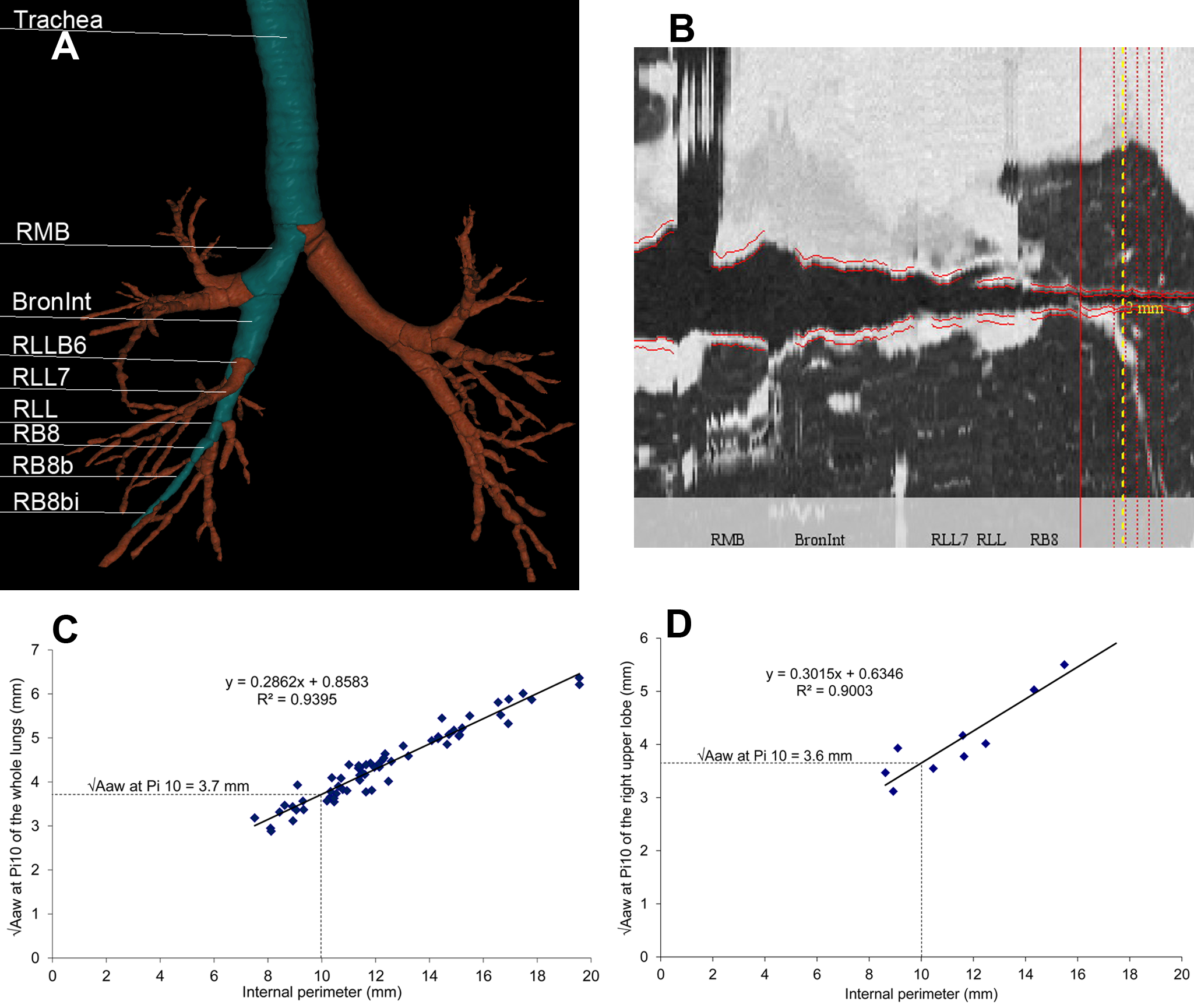

Supplement: Figure S2 — Airway analysis using Pulmonary Workstation 2. (A) The 3-D bronchial tree is segmented, and one bronchial pathway is labeled until the 5th generation (RMB, right main bronchus; BronInt, bronchus intermedius; RLL, right lower lobe basal bronchus; RB8, right basal anterior segmental bronchus–the 3rd generation; RB8b, right basal anterior sub-segmental bronchus–the 4th generation; RB8bi, right basal anterior sub-subsegmental bronchus–the 5th generation). (B) One bronchial pathway is reconstructed as a straightened airway image. The yellow dash line indicates where a 2-D slice, which is perpendicular to the centerline, is resampled for measuring airway dimensions at a centerline voxel position. (C) The square root of airway wall area of the hypothetical airway with an internal perimeter of 10 mm (√Aaw at Pi10) is derived from all measurable bronchial segments of the whole lungs of a representative COPD patient. (D) √Aaw at Pi10 is derived from all measurable bronchial segments of the right upper lobe of the same patient as in Panel C. (TIF) [file pone.0098335.s002.tif]

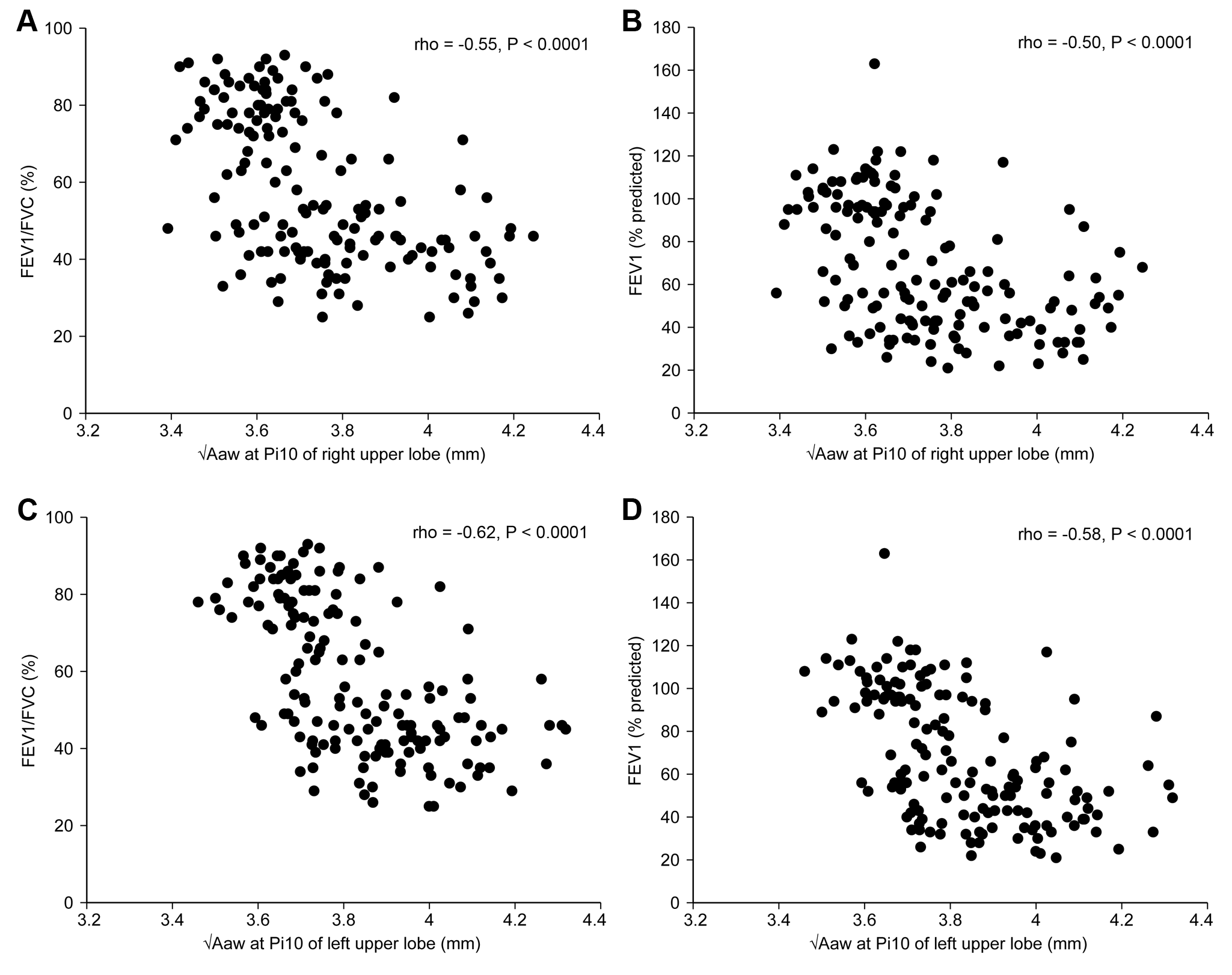

Supplement: Figure S3 — Correlations between √Aaw at Pi10 derived from the upper lobes and FEV1/FVC or FEV1% predicted. √Aaw at Pi10, square root of airway wall area of the hypothetical airway with an internal perimeter of 10 mm; FVC, forced vital capacity; FEV1, forced expiratory volume in one second. √Aaw at Pi10 derived from the right upper lobe is negatively associated with FEV1/FVC (Panel A) and FEV1% predicted (Panel B). Similarly, √Aaw at Pi10 derived from the left upper lobe is negatively associated with FEV1/FVC (Panel C) and FEV1% predicted (Panel D). (TIF) [file pone.0098335.s003.tif]
