# Supplementary material for: Airway Wall Area Derived from 3-Dimensional Computed Tomography Analysis Differs among Lung Lobes in Male Smokers
Source: PLoS One. 2014 May 27;9(5):e98335. doi: 10.1371/journal.pone.0098335 (PMC4035347; doi:10.1371/journal.pone.0098335)
Supplement: Table S1 — Coefficients of determination (R2) of the linear regression analyses for √Aaw at Pi10. (DOC) [file pone.0098335.s004.doc]

**Table S1.** Coefficients of determination (R^2^) of the linear regression analyses for Aaw at Pi10.

| **Lung lobe** | **R^2^** |
| --- | --- |
| Right upper | 0.95 (0.93, 0.96) |
| Right middle* | 0.94 (0.90, 0.97) |
| Right lower | 0.94 (0.91, 0.95) |
| Left upper | 0.94 (0.90, 0.96) |
| Left lower | 0.93 (0.90, 0.96) |
| Whole lungs | 0.92 (0.90, 0.94) |

Data are presented as medians (25^th^, 75^th^ percentiles).

√Aaw at Pi10, square root of airway wall area of the hypothetical airway with an internal perimeter of 10 mm

^*^data from 146 subjects
