# Supplementary material for: Airway Wall Area Derived from 3-Dimensional Computed Tomography Analysis Differs among Lung Lobes in Male Smokers
Source: PLoS One. 2014 May 27;9(5):e98335. doi: 10.1371/journal.pone.0098335 (PMC4035347; doi:10.1371/journal.pone.0098335)
Supplement: Text S1 — Details about the 3-D CT analysis using Pulmonary Workstation 2 software. (DOC) [file pone.0098335.s005.doc]

**Text S1.** Details about the 3-D CT analysis using Pulmonary Workstation 2 software.

**Emphysema analysis**

The emphysema analysis could be summarized in the following steps. Step 1: From the original CT scan (Figure S1, Panel A), the lungs were segmented and the right and left lungs were differentiated by the software. The observers edited the lung boundaries if necessary. Step 2: The five lung lobes were segmented using information from the labeled airway tree, feeding vessels, and lobar fissures (Figure S1, Panel B). Because a significant amount of lobar tissue needs to be exchanged before the extent of emphysema is significantly affected, the observers edited the lung lobes only if significant parts of the lobe structures were incorrectly assigned. Step 3: The software masked all pixels (Figure S1, Panel C) or voxels (Figure S1, Panel D) with CT attenuation less than –950 Hounsfield units. Then the software automatically generated the results of lung densitometry that included percentages of low attenuation volume (LAV%) for the whole lungs and for each individual lobe.

**Airway analysis**

The airway analysis could be summarized in the following steps. Step 1: The 3-D bronchial tree was automatically segmented by the software. Step 2: The observers checked all bronchial pathways using axial, sagittal, and coronal sections. If there were any visible bronchial segments until the 6^th^ generation that the software had not masked, the observers would set a seed point inside that bronchial segment and prompt the software to grow that bronchial segment. Only bronchial segments connected to the masked proximal bronchial segments were accepted. The observers repeated this step until all visible bronchial segments from the 3^rd^ to 6^th^ generations were masked. There are two reasons why we tried to segment the bronchial segment until the 6^th^ generation: first, to make sure visible bronchial segments with the 5^th^ generation intact in length; second, to avoid unnecessarily segmenting bronchial segments with internal perimeter less than 6 mm or to save time. Step 3: The software automatically labeled all bronchial segments until the 3^rd^ generation (segmental bronchi). The observers corrected these labels and labeled all masked bronchial segments until the 5^th^ generation (Figure S2, Panel A). The 3-D bronchial tree with proper labeling must be reconstructed before any measurements of airway dimensions. Step 4: For each bronchial pathway, the software reconstructed it to obtain the straightened airway (Figure S2, Panel B). From the straightened airway, the software could obtain the 2-D slices that run perpendicularly to the centerline. This is not feasible if 2-D CT analysis software is used because the airways do not always run perpendicularly to the axial sections. For each bronchial segment, the software automatically measured the airway dimensions—including the internal perimeter, inner area, and outer area—at every centerline voxel position along the middle third of that bronchial segment. For each airway dimension, the average of these measurements was the value of that bronchial segment. The airway wall area of a certain bronchial segment was calculated by subtracting the inner area from the outer area. Step 5: The observers selected results of all measurable bronchial segments from the 3^rd^ to 5^th^ generations with internal perimeters ranging from 6 mm to 20 mm for the whole lungs and separated them for each lobe using the Data Sort function of the Excel software. Step 6: The observers plotted the square root of the airway wall area against the internal perimeter of all measurable bronchial segments of the whole lungs or of each individual lobe using the Excel software. From the linear regression equation, the square root of airway wall area of the hypothetical airway with an internal perimeter of 10 mm (√Aaw at Pi10) was calculated for the whole lungs (Figure S2, Panel C) and for each individual lobe (Figure S2, Panel D).

We selected only bronchial segments with internal perimeter ranging from 6 mm to 20 mm for estimating √Aaw at Pi10 because of the following reasons. First, majority of bronchial segments from the 3^rd^ to 5^th^ generations have internal perimeters ranging from 6 mm to 20 mm. Second, the Pulmonary Workstation 2 software has been validated on physical phantoms (Plexiglas tubes) with known internal diameter equal to or more than 1.98 mm—internal perimeter more than 6 mm. Third, because we want to estimate the airway wall area of a hypothetical airway with an internal perimeter of 10 mm from the regression line, we do not need to measure bronchial segments with internal perimeter more than 20 mm.
